# Impact of Glyphosate on the Development of Insulin Resistance in Experimental Diabetic Rats: Role of NFκB Signalling Pathways

**DOI:** 10.3390/antiox11122436

**Published:** 2022-12-09

**Authors:** Monisha Prasad, Mansour K. Gatasheh, Mohammad A. Alshuniaber, Rajapandiyan Krishnamoorthy, Ponnulakhmi Rajagopal, Kalaiselvi Krishnamoorthy, Vijayalakshmi Periyasamy, Vishnu Priya Veeraraghavan, Selvaraj Jayaraman

**Affiliations:** 1 Centre of Molecular Medicine and Diagnostics (COMManD), Department of Biochemistry, Saveetha Institute of Medical & Technical Sciences, Saveetha Dental College & Hospitals, Saveetha University, Chennai 600077, India; 2 Department of Biochemistry, College of Science, King Saud University, Riyadh 11451, Saudi Arabia; 3 Department of Food Science and Nutrition, College of Food and Agriculture Sciences, King Saud University, Riyadh 11451, Saudi Arabia; 4 Department of Central Research Laboratory, Meenakshi Ammal Dental College and Hospitals, Chennai 600095, India; 5 Department of Biotechnology and Bioinformatics, Holy Cross College, Trichy 620002, India

**Keywords:** glyphosate, type 2 diabetes, GLUT2 expression, NFκB signalling pathways, rats

## Abstract

Glyphosate, an endocrine disruptor, has an adverse impact on human health through food and also has the potential to produce reactive oxygen species (ROS), which can lead to metabolic diseases. Glyphosate consumption from food has been shown to have a substantial part in insulin resistance, making it a severe concern to those with type 2 diabetes (T2DM). However, minimal evidence exists on how glyphosate impacts insulin-mediated glucose oxidation in the liver. Hence the current study was performed to explore the potential of glyphosate toxicity on insulin signaling in the liver of experimental animals. For 16 weeks, male albino Wistar rats were given 50 mg, 100 mg and 250 mg/kg b. wt. of glyphosate orally. In the current study, glyphosate exposure group was linked to a rise in fasting sugar and insulin as well as a drop in serum testosterone. At the same time, in a dose dependent fashion, glyphosate exposure showed alternations in glucose metabolic enzymes. Glyphosate exposure resulted in a raise in H_2_O_2_ formation, LPO and a reduction in antioxidant levels those results in impact on membrane integrity and insulin receptor efficacy in the liver. It also registered a reduced levels of mRNA and protein expression of insulin receptor (IR), glucose transporter-2 (GLUT2) with concomitant increase in the production of proinflammatory factors such as JNK, IKKβ, NFkB, IL-6, IL-1β, and TNF-α as well as transcriptional factors like SREBP1c and PPAR-γ leading to pro-inflammation and cirrhosis in the liver which results in the development of insulin resistance and type 2 diabetes. Our present findings for the first time providing an evidence that exposure of glyphosate develops insulin resistance and type 2 diabetes by aggravating NFkB signaling pathway in liver.

## 1. Introduction

Diabetes mellitus (DM) has been estimated to affect 578 million people worldwide in next few years, rising to 700 million by the 2045. It is a serious, chronic condition that adversely affects people, families, and societies worldwide. Its primary features include chronic hyperglycemia, a total or partial impairment in insulin action, and abnormalities in the metabolism of proteins, carbohydrates, and lipids [1]. Diabetes is a metabolic condition that is greatly influenced by nutrition, genetics, and environment. The presence of certain environmental pollutants may further increase the risk of developing diabetes. Herbicides are weed-control substances (insecticides) that have the potential to cause substantial environmental damage [2,3]. Because there is a strong relationship between these chemical molecules and diseases, particularly metabolic disorders, they have both good and detrimental impact on humans [4]. People are typically exposed to a variety of herbicides through the use of fresh fruits and vegetables as weed switch plant goods [5]. Herbicides are absorbed by the gastrointestinal/respiratory system and also via skin. These herbicides can metabolise in human organs, mainly in the liver tissue, due to their strong affinity and stability. They have a long persistence in the environment and because of their high toxicity and stability, they pollute; therefore, they require special attention these days with regards to human toxicity that results in the development of diabetes [6].

The liver is a vital organ for the metabolism of energy as well as the detoxification of xenobiotics, environmental hormones, and hazardous chemicals. The inhibition of gluconeogenesis and enhanced storage of glucose as glycogen are brought on by insulin signalling, particularly in hepatocytes. Additionally, NF-κB, a DNA-binding protein factor, is involved in the transcription of many pro-inflammatory and inflammatory molecules, including cytokines, chemokines, cell adhesion molecules (CAM), and various enzymes [7]. Specifically, in metabolic organs such as liver muscle and adipose tissue, the expression of cytokines and inflammatory molecules have a substantial impact on the aetiology of diabetes. Examples include compromised insulin signalling in hepatocytes, elevated free fatty acids, systemic insulin resistance, and glucose intolerance due to constitutively active (CA) IKK expression in the liver brought on by these hazardous substances or the metabolic imbalance [8]. Inflammation and insulin resistance are caused by environmental factors such as toxins or other modulators that can impact metabolic signalling gene expression and enhance NFκB signalling via proinflammatory cytokines [9].

Glyphosate is one of the phosphonate and systemic herbicide used globally to control weed in farming and agriculture [10]. It is marketed in the brand names “Roundup” and “Ranger Pro”, and it’s often assorted with supposedly innocuous ingredients like surfactants. This herbicide, which comes in a variety of salt forms impedes the conversion of shikimate-3-phosphate to 5-endopyruvylshimikate 3-phosphate by 5-enolpyruvylshikimate-3-phosphate synthase (shikimic acid pathway), thereby hampering the aromatic amino acids production such as tyrosine, phenylalanine and tryptophan [11]. This shikimic acid pathway, which is exclusive to plants, bacteria, and fungi, is consequently eliminated by glyphosate. Glyphosate, being a chelating agent interferes with nutrients required for a variety of plant processes, has the potential to alter plant metabolism, which also has implications for humans. Ingestion of glyphosate orally through food, drinking water and other ecological channels has been associated to endocrine disruption, cancer, mutagenicity and reproductive damage [12,13,14].

Previous studies have shown that glyphosate is hazardous to the reproductive system particularly, the exploration of glyphosate has numerous adverse consequences on the male reproductive system in both humans and experimental animal models [15]. Furthermore, it was found to be a strong human endocrine disruptor that affects serum testosterone and is subsequently linked to higher glucose and insulin levels, obesity, the distribution of fat in the upper body, and is strongly linked to insulin resistance and the other metabolic syndrome [16]. Additionally, insulin resistance is linked to the castration of male rats, and the condition is corrected with subsequent testosterone replacement, demonstrating the connection between testosterone insufficiency and diabetes [17]. As instance, Muthusamy et al. [18] established that insulin and its receptor gene expression were disrupted in testosterone deficiency-induced experimental animals that resulted in impaired glucose oxidation in skeletal muscles, liver, and adipose tissue. In addition to its effects on endocrine function, glyphosate exposure in experimental animals results in hyperglycemia, reactive oxygen species (ROS), and related metabolic problems [19,20,21,22]. Despite these findings, it is unclear what mechanisms underlie glyphosate’s particular effects on insulin signalling and glucose homeostasis in hepatocytes. This work aims to investigate into the mechanism of glyphosate-induced toxic effects on insulin signalling in rat liver.

## 2. Materials and Methods

### 2.1. Chemicals

All chemicals and reagents used in the present study were of molecular and analytical grade; and they were purchased from Sigma Chemical Company, Krishgen Biosystem, DBC Diagnostics Biochem, Invitrogen, New England Biolabs (NEB), Eurofins Genomics and Santa Cruz Biotechnology. Glyphosate (Product no. 45521) was purchased from Sigma Chemical Company, St. Louis, MI, USA. ELISA kits for insulin, IL-6 and IL-1 were bought from Krishgen Biosystem, Mumbai, India. INS GENLISA ELISA kit was bought from Krishgen Biosystem, Mumbai, India and testosterone ELISA kit from DBC Diagnostics Biochem, London, ON, Canada. The total RNA isolation kit was supplied by Invitrogen, USA. The RT enzyme was provided by New England Biolabs (NEB), Ipswich, MA, USA and the Go Taq Green master mix was provided by Promega, Madison, WI, USA. The primers for sterol regulatory element-binding transcription factor 1 (SREBP1c), interleukins (IL-6, IL-1β), insulin receptor (IR), Inhibitory kappaB kinase beta (IKKβ), c-Jun N-terminal kinases (JNK), peroxisome proliferator-activated receptor gamma (PPAR-γ), glucose transporter-2 (GLUT2), tumour necrosis factor (TNF-α), Nuclear factor kappa B (NFκB) and β-actin were supplied by Eurofins Genomics India, Pvt. Ltd., Bangalore, India. TNF-α and IL-6 antibodies were provided by Santa Cruz Biotechnology, Santa Cruz, CA, USA.

### 2.2. Animals and Induction of Diabetes with Glyphosate

Eight week old adult male Wistar albino rats (*Rattus norvegicus*) weighing 180–200 g were used in the present study. Rats were maintained under the standard environmental conductions as per the guidelines of institutional animal ethics committee (IAEC) with temperature (24 ± 2 °C), 50% humidity and continual 12 h dark and 12 h light cycle. They were fed with standard pellet and water ad libitum. The study was approved (IAEC No. BRULAC/SDCH/SIMATS/IAEC/02-2019/015) by Biomedical Research Unit and Laboratory Animal Centre (BRULAC), Saveetha Dental College and Hospitals, SIMATS, Chennai-77 and the study conducted in accordance with the “Guide for the care and use of laboratory animals”.

Glyphosate dose selection in the present study was based on the previous reports [23,24]. Glyphosate at the dose of 50, 100 and 250 mg/kg b.wt was dissolved in water and treated orally. The experiment lasted for 16 weeks during which the fasting blood glucose levels of the experimental group were greater than 200 mg/dL were served as diabetic rats.

### 2.3. Experimental Time Line

Rats were randomly divided into four groups of six rats each.

Group 1. Normal control rats treated with vehicle (water only)
Group 2. Normal rats received oral administration of glyphosate dissolved in water (50 mg/kg b. wt./day) for 16 weeks.
Group 3. Normal rats received oral administration of glyphosate dissolved in water (100 mg/kg b. wt./day) for 16 weeks.
Group 4. Normal rats received oral administration of glyphosate dissolved in water (250 mg/kg b. wt./day) for 16 weeks.

On the last day of experiment the animals in various groups were sacrificed by cervical decapitation under sodium thiopentone (40 mg/kg b. wt.) anesthesia. The blood was drawn via venipuncture and the sera was separated by centrifugation and then stored at −80 °C. 20 mL of isotonic NaCl solution was injected into the left ventricle to remove blood from various parts of the body [25]. The liver was promptly dissected to be used in the experiment immediately.

### 2.4. Determination of Fasting Sugar, Serum Insulin and Testosterone

The animals were starved overnight the day before sacrifice after receiving glyphosate treatment for 16 weeks. The blood was drawn the next day from the rat tail tip to estimate the fasting blood glucose (FBG) levels using On-Call Plus blood glucose test strips. The data were displayed in milligrammes per decilitre on the metre display window.

ELISA technique was used to measure the levels of insulin and testosterone in rat serum. As per instruction provided in the manual, the detection range and coefficient of variation were set. The testosterone concentration was measured in ng/mL and the serum insulin concentration was measured in pg/mL.

### 2.5. Determination of Oral Glucose Tolerance (OGT)

For OGT, the control and glyphosate-exposed rats remained fasted overnight. After an oral glucose load (10 mL/kg; 50% *w*/*v*), we monitored fasting blood glucose level using glucose test strips at 60-min, 120-min and 180-min time intervals. The glucose level was measured as the 0-min value prior to administration of glucose. The values were measured in mg/dL.

### 2.6. Determination of Insulin Tolerance (IT)

Insulin (0.75 U/kg b.wt. i.p.) was given to each group two days before the animals were slaughtered. At 0 h, the level of blood glucose were tested at different time intervals. The results were given in mg/dL of blood.

### 2.7. HOMA-IR and QUICKI

The insulin sensitivity index was investigated using the HOMA-IR and QUICKI calculation methods developed by Matthews et al. [26] and Katz et al. [27]. The equations fasting blood glucose+fasting serum insulin/405 and 1/log fasting serum insulin+log fasting blood glucose, respectively, were used to calculate HOMA-IR and QUICKI.

### 2.8. Measurement of Oxidative Stress Marker

Using the methodologies of Fraga et al. [28] and Jiang et al. [29], thiobarbituric acid reactive substances (TBARS) and lipid peroxidation (LPO) in the tissue were evaluated calorimetrically. The results were represented in mM/mg tissue.

### 2.9. Measurement of Antioxidant Enzymes

Superoxide dismutase (SOD) was quantified using the procedure mentioned in Kakkar et al. [30] and the findings were expressed as units/mg protein. Sinha’s method [31] was used to quantify the catalase activity (CAT), and expressed as units/mg protein.

### 2.10. Determination of Specific Enzymes Involved in Carbohydrate Metabolism

Hexokinase (HK) activity was measured using the method of Brandstrup et al. [32]. The enzyme’s activity was calculated in mol glucose phosphorylated/h/mg protein. The enzymatic activity of pyruvate kinase (PK) in tissues was measured using Valentine and Tanaka’s [33] technique. The results were expressed as μmol pyruvate formed/min/mg protein.

The activity of hepatic glucose-6-phosphatase (G6P) was examined using the Koida and Oda’s technique [34]. The G6P activity was measured in moles of inorganic phosphorus liberated/min/mg protein. Gancedo and Gancedo’s [35] method was used to measure the activity of hepatic fructose-1,6-bisphosphatase (F1,6BP). The activity of the enzyme was measured in moles of inorganic phosphorus liberated/hh/mg protein.

### 2.11. Total RNA, cDNA Synthesis and Real-Time PCR

The total RNA was isolated from the liver of the rats according to the procedure described in Fourney et al. [36]. About 2 µg of RNA was taken and reverse transcribed into cDNA using the RT kit (Seraing, Belgium). The primers utilized in this study are listed in Table 1. The SYBR green mastermix (Takara, Japan) and housekeeping gene (β-actin) were used in a Real-Time PCR system (Bio-Rad C1000 Touch, thermal cycler, Bio-Rad Laboratories Ltd. (Bio-Rad House, Herts, UK) to amplify the interest genes under the following reaction conditions: initial denaturation at 95 °C for 5 min, followed by 40 cycles of denaturation: 95 °C for 30 s, annealing: 60 °C for 1 min and extension: 72 °C for 30 s each. The analyses of melt and amplification curves were carried out to calculate the relative quantification.

### 2.12. Protein Analysis of Pro-Inflammatory Cytokines and Transcription Factors

Commercial rat ELISA kits purchased from Krishgen Biosystem, Mumbai 400018, India (for serum IL-6 and IL-1β), Ray Biotech, Illinois, GA, USA (for TNF-α) and My BioSource, Inc., San Diego, CA, USA (transcription factors such as PPAR-γ and SREBP1c) were used to measure the protein concentration in experimental rats as directed by the manufacturer’s manual. SREBP-1c, IL-1β, IL-6 and TNF-α levels were measured in pg/mL whereas PPAR-γ levels were measured in ng/mL.

### 2.13. Histopathological Staining

By embedding the liver tissue in 10% neutral buffered formalin, followed by sectioning and staining it with hematoxylin and eosin dye, the morphology of the liver was studied [45,46]. Then, using a LKB ultra-microtome, semi-thin sections (0.5–1 µ) were cut, stained with toluidine blue and shot using an Olympus light microscope and a Nikon digital camera at a magnification of X200.

### 2.14. Immunohistochemical Staining

Immunohistochemistry was performed on paraffin embedded liver slices of control and glyphosate-treated rat tissues using a microwave-based antigen retrieval technique. The segments were incubated overnight at 4 °C in a dark, humid chamber with primary polyclonal anti-TNF-α and anti-IL-6 (1:100 dilution) antibodies, followed by 45 min incubation with secondary antibodies under the same conditions. After that, the slides were washed in 1 M PBS to eliminate any remaining secondary antibodies and incubated with horse-radish peroxidase (HRP) for 45 min in a humidified environment before being rinsed for 5 min in 1 M PBS. Then, the slides were incubated for 6 min at 37 °C with the 3,3′-diaminobenzidine (DAB) substrate chromogen 3.3, followed by a 5-min wash in water. The specimens were dried, cleaned and mounted after being counterstained with hematoxylin for 40 s. To ensure the quality of the staining, positive and negative controls were used for each immunohistochemical staining procedure. To establish the overall distribution of the main protein, the cells were initially examined at a modest magnification 100×).

### 2.15. Statistical Analysis

Using computer-based software, the significance difference between the control group and treatment sets was assessed by one-way analysis of variance (ANOVA) and Duncan’s multiple range tests using computational software Graph Pad Prism version 5. The significance of Duncan’s test was determined at the level of *p* < 0.05.

## 3. Results

### 3.1. Impact of Glyphosate on FBG, Insulin and Testosterone

**Figure 1:**
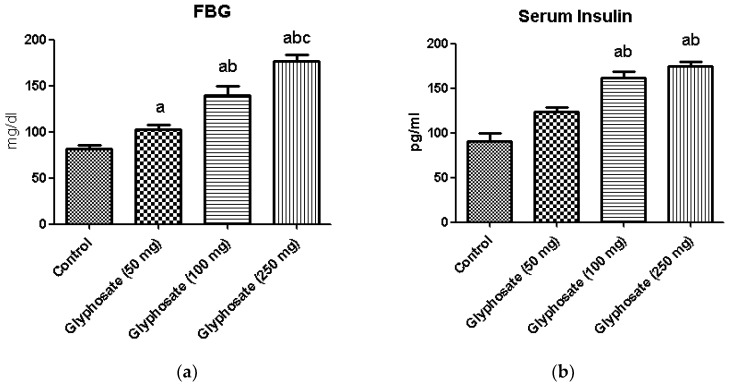

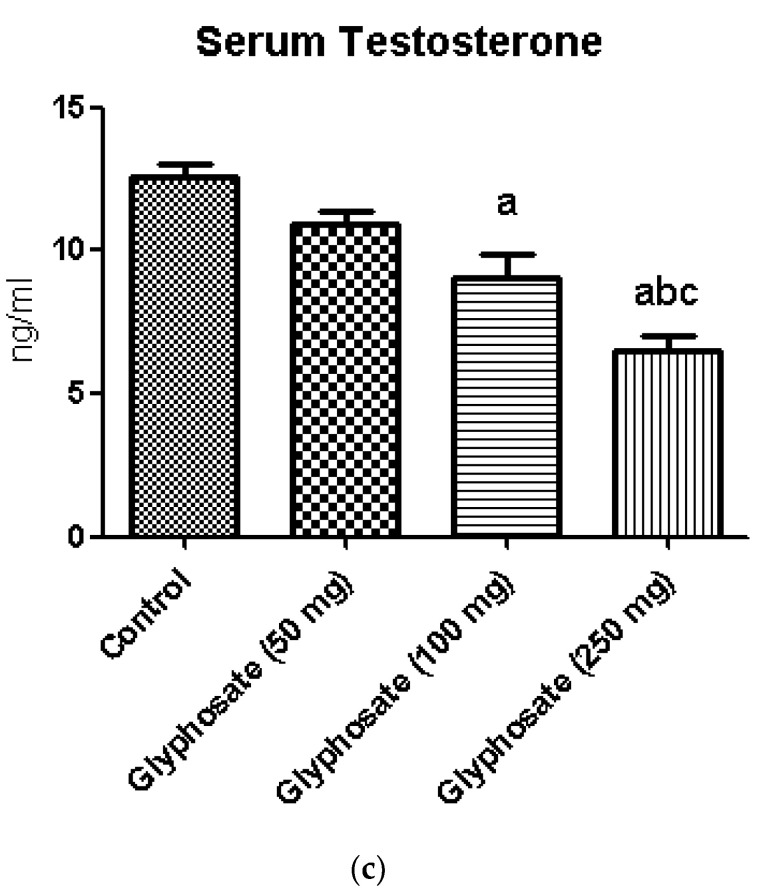
(**a**–**c**): The effect of glyphosate on FBG (**a**), serum insulin (**b**) and the serum testosterone (**c**) in control and glyphosate treated rats. Data were expressed as mean ± SEM where *n* = 6 rats for each group. Values significant at *p* < 0.05 were analyzed with a—control, b—glyphosate (50 mg/kg b.wt.), and c—glyphosate (100 mg/kg b.wt.).

Fasting blood glucose (Figure 1a) and serum insulin (Figure 1b) were significantly raised in the glyphosate exposed groups compared to control. Conversely, serum testosterone concentration (Figure 1c) was found to be reduced in a dose dependent manner. These results clearly indicate that glyphosate has the toxic effect at 50–250 mg/kg b.wt. dose to induce hyperglycemia and insulin resistance in 16 weeks duration.

### 3.2. Impact of Glyphosate on OGT

Table 2 depicts the OGT levels in experimental and control rats with time. Blood glucose levels increased after taking glucose orally; this rise is dose-dependent and peaks at one hour. At 180 min, the glucose level in control rats had returned to normal (76 mg/dL), whereas it was significantly higher in the glyphosate-induced groups, demonstrating that the glyphosate had caused glucose intolerance.

### 3.3. Impact of Glyphosate on IT

Table 3 shows the ITT levels of normal and diabetic (Glyphosate treated) rats after oral administration of insulin (0.75 U/kg b.wt. i.p.). In diabetic control rats, the highest increase in serum insulin level was seen after 30 min and it was persisted high for over an hour has caused changes in insulin function.

### 3.4. Impact of Glyphosate on HOMA-IR and QUICKI

Table 4 illustrates the HOMA-IR and QUICKI calculations for rats exposed to glyphosate and rats in the control group. HOMA-IR was significantly increased in glyphosate-induced diabetic rats compared to control rats, whereas QUICKI values decreased. These significant differences in the insulin-sensitivity check index between normal and diabetic rats show that glyphosate impacts insulin signaling, which in turn affects glucose metabolism and leads to glucose and insulin intolerances.

### 3.5. Impact of Glyphosate on Oxidative Stress Markers

**Figure 2:**
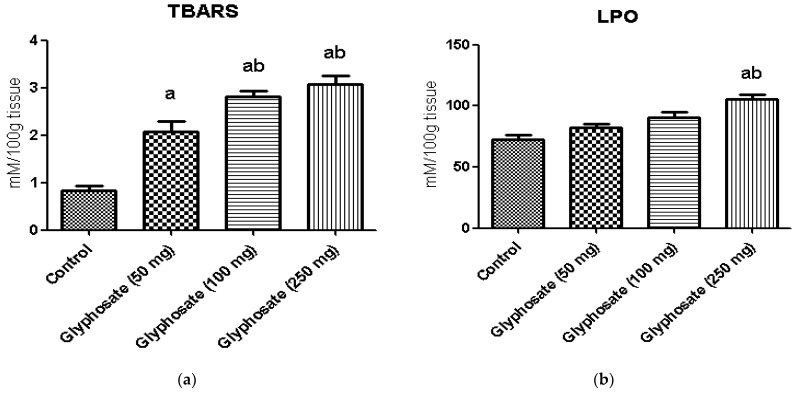
(**a**,**b**): The effect of glyphosate on oxidative stress markers (TBARS and LPO) in control and glyphosate treated rats. Data were expressed as mean ± SEM where *n* = 6 rats for each group. Values significant at *p* < 0.05 were analyzed with a—control, b—glyphosate (50 mg/kg b.wt.).

The changes in the levels of hepatic thiobarbituric acid (TBARS) and lipid peroxidation (LPO) in control and experimental rats are shown in Figure 2a,b. The levels of thiobarbituric acid reactive substances, and lipid peroxidation were significantly increased (*p* < 0.05) in glyphosate-treated rats when compared with normal control rats.

### 3.6. Impact of Glyphosate on SOD and CAT Enzymes Activity

**Figure 3:**
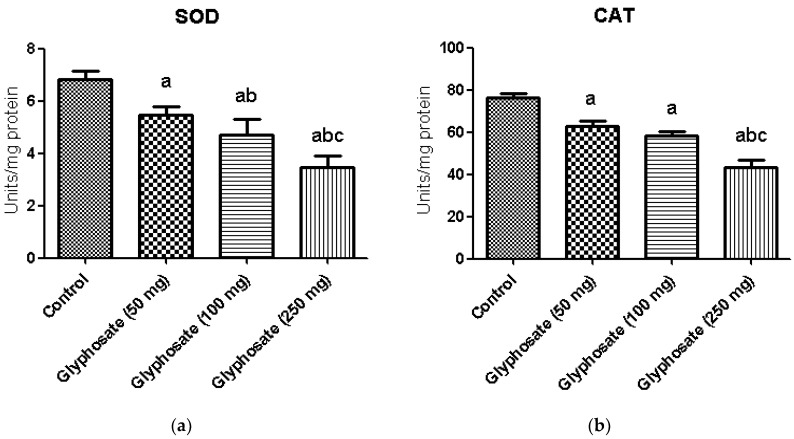
(**a**,**b**): The effect of glyphosate on antioxidant enzymes (SOD and CAT) in control and glyphosate treated rats. Data were expressed as mean ± SEM where *n* = 6 rats for each group. Values significant at *p* < 0.05 were analyzed with a—control, b—glyphosate (50 mg/kg b.wt.), and c—glyphosate (100 mg/kg b.wt.).

Figure 3a,b illustrates the activities of enzymatic antioxidants namely superoxide dismutase (SOD) and catalase (CAT) in the liver of control and experimental rats. A significant (*p* < 0.05) depletion in the activities of enzymatic antioxidants in glyphosate treated rats was observed. Treatment of glyphosate decreased the levels of these enzymatic antioxidants in the liver indicate its toxicity.

### 3.7. Impact of Glyphosate on Glycolytic and Gluconeogenic Enzymes

**Figure 4:**
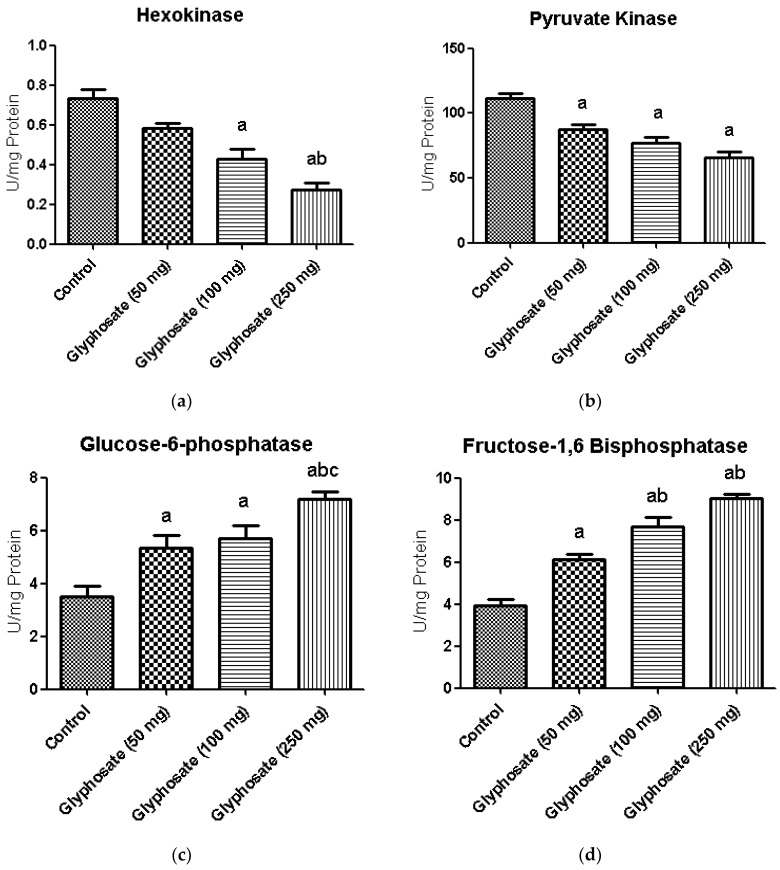
(**a**–**d**): The effect of glyphosate on the activity of glycolytic (hexokinase and pyruvate kinase) and gluconeogenic (glucose-6-phosphatase and fructose-1,6-bisphosphatase) enzymes in control and glyphosate treated rats. Data were expressed as mean ± SEM where *n* = 6 rats for each group. Values significant at *p* < 0.05 were analyzed with a—control, b—glyphosate (50 mg/kg b.wt.), and c—glyphosate (100 mg/kg b.wt.).

Figure 4a–d depicts the activities of carbohydrate metabolizing enzymes in liver of normal and glyphosate-treated diabetic rats. The activities of glycolysis enzyme hexokinase and pyruvate kinase was found to be decreased whereas the activities of gluconeogenic enzymes like glucose-6-phosphatase and fructose-1,6-bisphosphatase were significantly increased in diabetic rats compared to normal rats.

### 3.8. Impact of Glyphosate on the mRNA Expression of Insulin Signalling Molecules

**Figure 5:**
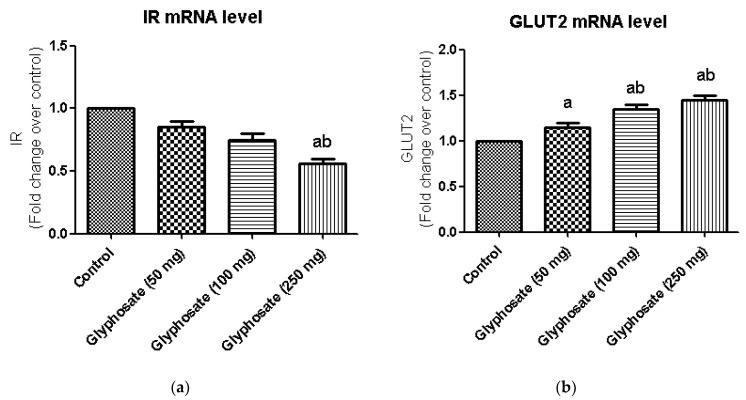
(**a**,**b**): The effect of glyphosate on mRNA levels of IR and GLUT2 in control and glyphosate treated rats. IR and GLUT 2 mRNA expression were analyzed by Real Time-PCR using gene specific primers and results were expression in fold-change over control. Data were expressed as mean ± SEM where *n* = 6 rats for each group. Values significant at *p* < 0.05 were analyzed with a—control, b—glyphosate (50 mg/kg b.wt.).

The glyphosate effect on IR and GLUT2 mRNA levels in the liver is depicted in Figure 5a,b. Insulin-stimulated signal transduction mechanisms for hepatic glucose generation, including insulin receptors and downstream mediators, have been reduced in hepatic insulin resistance. We found a significant decrease in IR and GLUT2 mRNA expression in glyphosate-induced rats in this study.

### 3.9. Impact of Glyphosate on the mRNA Expression of Transcriptional Factors Like PPAR-γ and SREBP1c

**Figure 6:**
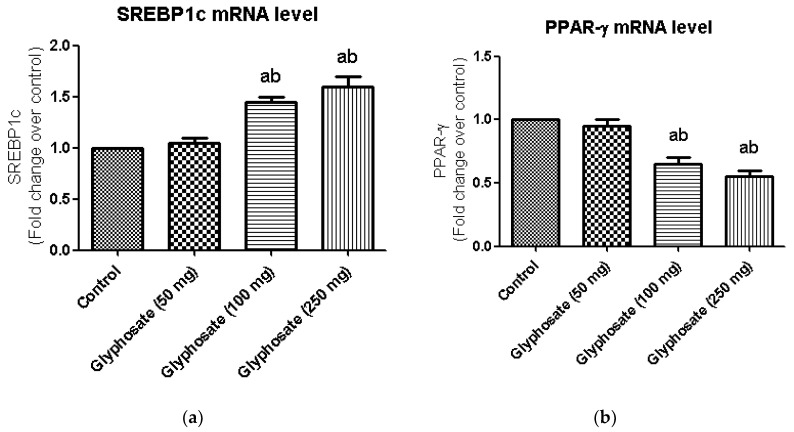
(**a**,**b**): Impact of glyphosate on mRNA levels of transcriptional factors like SREBP1c and PPAR-γ in control and glyphosate treated rats. SREBP1c and PPAR-γ mRNA expression were analyzed by Real Time-PCR using gene specific primers and results were expression in fold-change over control. Data were expressed as mean ± SEM where *n* = 6 rats for each group. Values significant at *p* < 0.05 were analyzed with a—control, b—glyphosate (50 mg/kg b.wt.).

Major transcriptional factors of hepatic insulin signalling like SREBP1c and PPAR-γ which commonly seen in the regulation of insulin signalling, were investigated using qRT-PCR (Figure 6a,b) in the present study. SREBP1c mRNA expression was significantly higher in glyphosate-treated diabetic rats (Figure 6a) when compare to control, although PPAR-γ mRNA expression was significantly reduced in experimental rats as compared to normal rats liver (Figure 6b).

### 3.10. Impact of Glyphosate on the mRNA Expression of JNK Pathway Related Molecules

**Figure 7:**
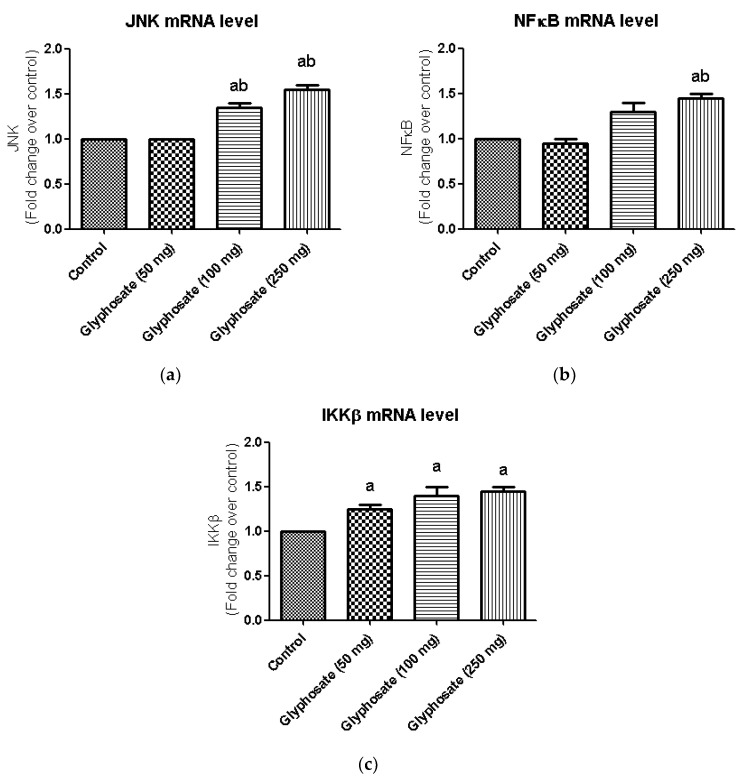
(**a**–**c**): Impact of glyphosate on the mRNA expression levels of JNK, NFκB and IKKβ in control and glyphosate treated rats. JNK, NFκB and IKKβ mRNA expression were analyzed by Real Time-PCR using gene specific primers and results were expression in fold-change over control. Data were expressed as mean ± SEM where *n* = 6 rats for each group. Values significant at *p* < 0.05 were analyzed with a—control, b—glyphosate (50 mg/kg b.wt.).

The JNK pathway is known to be active in diabetes and may have a role in the evolution of diabetes. JNK, NFκB and IKKβ mRNA levels were evaluated in the liver of glyphosate-treated rats. In glyphosate exposed rats, the expression of JNK, NFκB and IKKβ signalling molecules was shown to be significantly higher (Figure 7a–c) indicating the glyphosate’s ability to induce inflammation.

### 3.11. Impact of Glyphosate on the mRNA Expression of Pro-Inflammatory Cytokines

**Figure 8:**
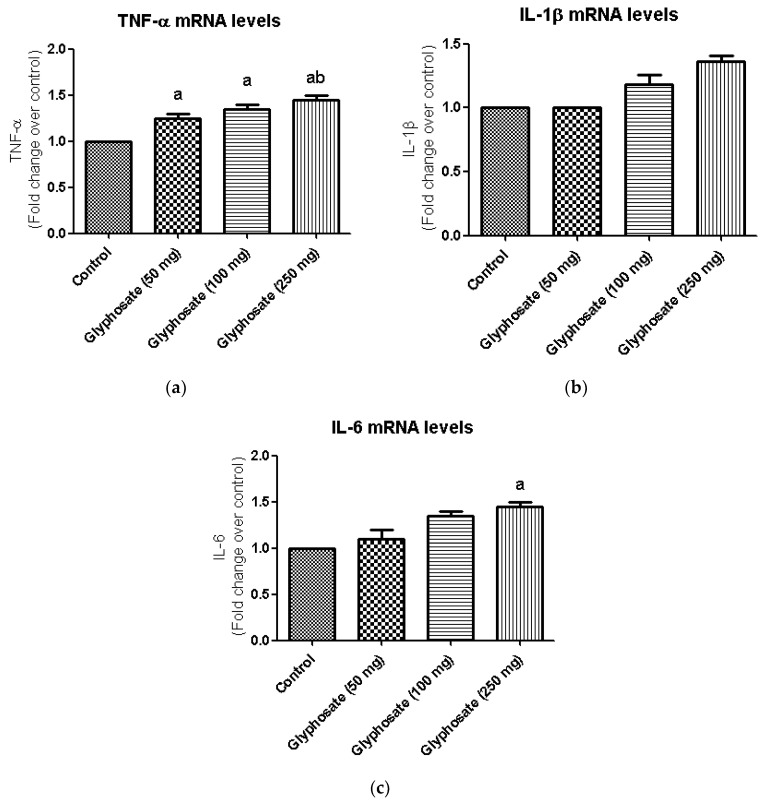
(**a**–**c**): The effect of glyphosate on mRNA levels of pro-inflammatory cytokines (TNF-α, IL-1β, and IL-6) in control and glyphosate treated rats. TNF-α, IL-1β, and IL-6 mRNA expression were analyzed by Real Time-PCR using gene specific primers and results were expression in fold-change over control. Data were expressed as mean ± SEM where *n* = 6 rats for each group. Values significant at *p* < 0.05 were analyzed with a—control, b—glyphosate (50 mg/kg b.wt.).

Inflammatory reactions are triggered by the production of pro-inflammatory cytokines such as TNF-α IL-1β, and IL-6 which are linked in the development of a number of metabolic diseases. Hence, we examine the mRNA expression levels of TNF-α, IL-1β, and IL-6 in glyphosate-treated group (Figure 8a–c). In this study, the hepatic mRNA expression levels of TNF-α and IL-6 were increased significantly (*p* < 0.05) in glyphosate-induced rats when compared to normal rats (Figure 8a,c); whereas, the IL-1β mRNA levels were unaltered in all the groups (Figure 8b).

### 3.12. Impact of Glyphosate on the Protein Analysis of Pro-Inflammatory Cytokines and Transcription Factors

**Figure 9:**
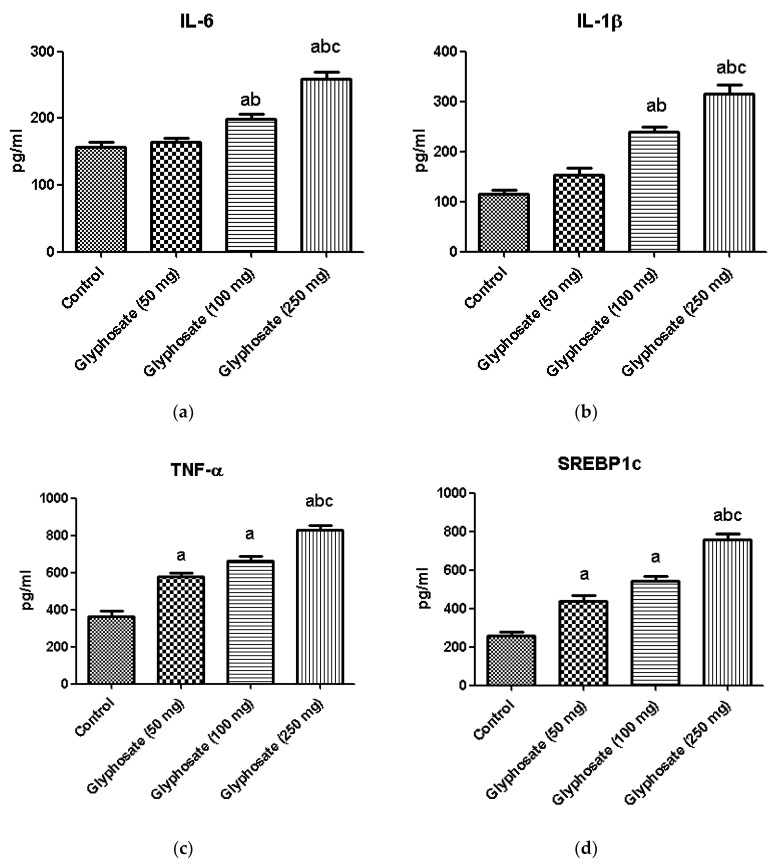

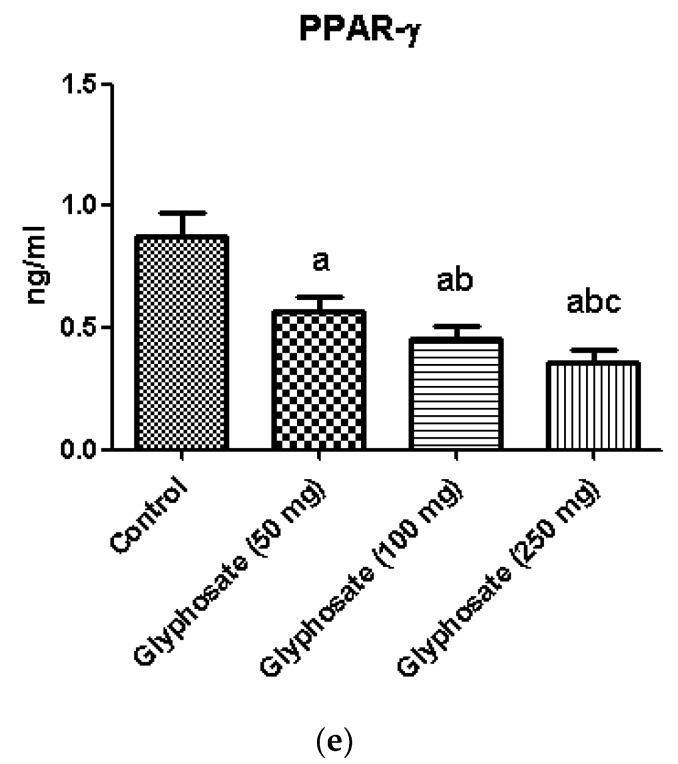
(**a**–**e**): The effect of glyphosate on protein levels of pro-inflammatory cytokines (IL-6, IL-1β, and TNF-α) and transcriptional factors (SREBP1c and PPAR-γ) in control and glyphosate treated rats. Data were expressed as mean ± SEM where *n* = 6 rats for each group. Values significant at *p* < 0.05 were analyzed with a—control, b—glyphosate (50 mg/kg b.wt.) and c—glyphosate (100 mg/kg b.wt.).

The development of diabetes is linked to chronic inflammation and regulation of many transcriptional factors. We used the ELISA technique to quantify the protein of proinflammatory cytokines like IL-6, IL-1β and TNF-α and the transcriptional factors SREBP1c and PPAR-γ (Figure 9a–c). When compared to the control rats, the glyphosate-treated group had considerably higher protein concentration of proinflammatory and transcriptional factors in glyphosate treated groups whereas the PPAR-γ level was significantly (*p* < 0.05) decreased showing that glyphosate caused low-grade liver inflammation (Figure 9d,e).

### 3.13. Impact of Glyphosate on the Expression of Pro-Inflammatory Proteins in Sections of the Liver Using Immunohistochemistry

**Figure 10:**
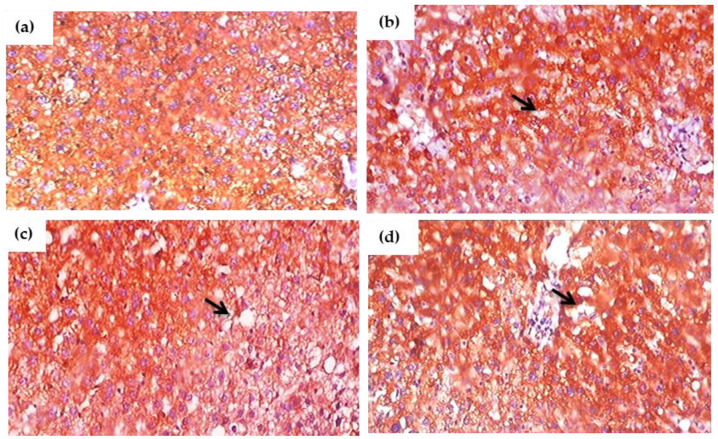
(**a**–**d**). Immunohistochemical evaluation of the effect of glyphosate on TNF-α protein expression in the liver sections of experimental rats. This image depicts exemplary IHC photomicrographs (100× magnification) of TNF-α staining intensity (black arrows) in the liver tissues. (**a**) Normal control rats showing small and large diameter hepatocytes; (**b**) glyphosate-exposed rats (50 mg/kg b.wt.) showed an mild expression of TNF-α with change in intensity that reflexed mild hydropic degeneration; (**c**) glyphosate-treated rats (100 mg/kg b.wt.) showed an elevated expression of TNF-α with increased hydropic degeneration indicated in the black arrow; and (**d**) glyphosate -treated rats (250 mg/kg b.wt.) showed an elevated expression of TNF-α with moderate expression and sever hydropic degeneration when compared to control rats.

**Figure 11:**
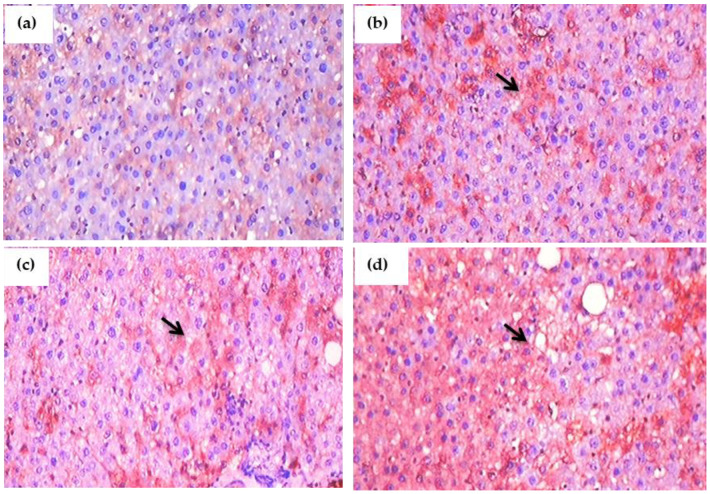
(**a**–**d**): Immunohistochemical evaluation of the effect of glyphosate on IL-6 protein expression in the liver sections of experimental rats. This image depicts typical IHC photomicrographs (100× magnification) of IL-6 staining intensity (black arrows) in the liver tissues of experimental group. (**a**) Normal control rats showing small and large diameter hepatocytes; (**b**) glyphosate-treated rats (50 mg/kg b.wt.) showed IL-6 expression with mild hydropic degeneration of hepatocytes; (**c**) glyphosate-treated rats (100 mg/kg b.wt.) showed elevated expression of IL-6 with moderate hydropic degeneration and focal necrosis; and (**d**) glyphosate-treated rats (250 mg/kg b.wt.) showed increased expression of IL-6 with high level of hydropic degeneration and focal necrosis when compared to control rats.

Figure 10a–d and Figure 11a–d depict the results of an immunohistochemical study of TNF-α and IL-6 in the liver tissues of rats exposed to glyphosate and control rats. When compared to control rats, the glyphosate-exposed group showed greater staining for inflammatory mediators such TNF-α and IL-6. Microscopic analysis of liver sections revealed light staining, indicating a weak expression of TNF-α and IL-6 in the samples from the normal group. The glyphosate-induced group of rat livers, on the other hand, showed a progressive increase in the mild to moderate expression of TNF-α and IL-6 along with an increase in high fluorescence intensity. This clearly indicates that glyphosate can cause mild to severe hydropic degeneration and focal necrosis as well as inflammation via TNF-α and IL-6.

### 3.14. Histopathological Observation

**Figure 12:**
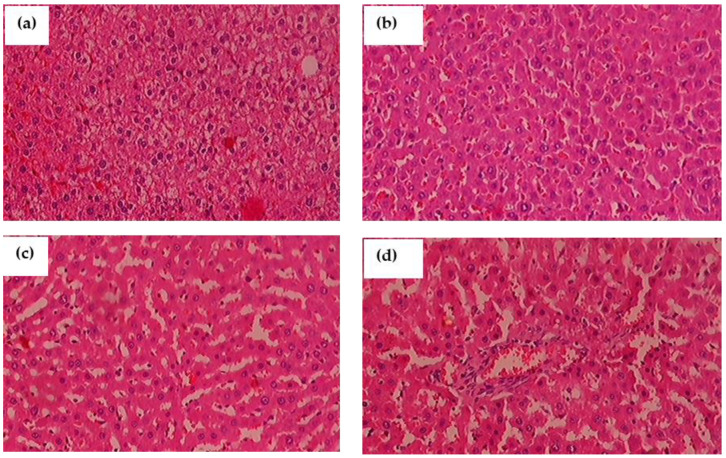
(**a**–**d**): The impact of glyphosate exposure on liver histopathology of experimental animals using hematoxylin and eosin (H&E) stain. (**a**) In normal rats, the hepatocyte nuclei are round and located inside the cells; (**b**) glyphosate-exposed rats (50 mg/kg b.wt.) showed a slight change in hepatocyte morphology when compared to control rats as the white patches around the nuclei depict lipid vesicles present inside the hepatocytes; (**c**) glyphosate-treated rats (100 mg/kg b.wt.) showed a significant fibrosis architecture of the liver with moderate lipid vesicles when compared to control rats; and (**d**) When compared to control rats, glyphosate (250 mg/kg b.wt.) rats showed elevated levels of liver fibrosis and inflammatory cells with large scale of white patches, demonstrating glyphosate toxicity in the liver.

The histological segments of the liver were examined with H&E staining is shown in Figure 12a–d. When compared to control rats, glyphosate-induced animals had an enlarged liver with more inflammation and fibrosis. Glyphosate exposed rats showed the change on hepatocyte morphology in the dose depended manner, as the 50 mg/kg b.wt. glyphosate explore showed the mild white patches around the nuclei (Figure 12b) compare to control with normal hepatocyte nuclei located inside the cells (Figure 12a). Glyphosate explore rats with 100 mg/kg b.wt. showed the microvesicular steatosis characterized by the presence of numerous small vesicles of fat that do not displace the nucleus (Figure 12c). There was the high number of large fat globule that displaces the nucleus as macrovesicular steatosis appears in Figure 12d.

## 4. Discussion

The most widely used herbicide in the world, glyphosate, has been linked to numerous environmental problems. Environmental exposure to glyphosate-based herbicides has recently been associated to hormone disturbance, kidney damage, liver toxicity, and other tissue damage at concentrations below regulatory boundaries. Additionally, these environmental pollutants activate NF-κB signalling, which plays a central role in the development of many metabolic diseases [46,47,48]. Therefore, the aim of the current study was to ascertain how glyphosate influence insulin signalling and glucose metabolism through NF-κB signalling in the liver of adult male albino rats.

Any substance that creates a toxic environment causes ectopic lipid build-up in the skeletal muscle and liver, obstructing insulin action. Following adipocyte failure, macrophage infiltration and increased lipolysis significantly affects hepatic lipid and glucose metabolism in a variety of ways. While this increases β cell function and hyperinsulinemia compensates for these dysregulated routes at first, glucolipotoxicity and genetic factors eventually contribute to β cell dysfunction and type 2 diabetes mellitus (T2DM) development [49]. Similarly, when comparing glyphosate-exposed rats to control rats, we discovered a significant increase in blood glucose and insulin levels. Furthermore, as an endocrine disruptor, glyphosate exposure affects testosterone production in rats, which was also seen in the current study. In previous studies, adult male rats treated with glyphosate had lower levels of blood testosterone and total sperm content, as well as more defective sperm [50,51]. This study demonstrated that exposure to glyphosate, affected testosterone levels through raising blood sugar and insulin levels which may be related to the generation of reactive oxygen species (ROS), which in turn affect these blood parameters.

In the current study, glyphosate-treated rats revealed an upsurge in blood glucose and serum insulin levels and these effects could be related to insulin resistance caused by the activation of hypothalamic-pituitary-adrenal (HPA) axis along with oxidative stress allied with hepatic insulin resistance [52,53]. Subsequently, rats exposed to glyphosate showed significant changes in the critical parameters such as OGT, ITT, HOMA-IR and QUICKI indexes in the current study, showing that glyphosate affects liver metabolism by creating ROS. In living systems, ROS-induced oxidative stress has both beneficial and harmful effects, including involvement in diabetogenesis and the development of diabetes associated complications [25,54].

ROS are produced by organophosphate herbicides like glyphosate, which leads to oxidative stress in the liver and other tissues, contributing to the toxic consequences of these xenobiotics. Oxidative stress-induced ROS can cause lipid peroxidation in the liver and brain, as well as damages DNA which in turn brings about tissue damage. Similarly, recent data on glyphosate-induced oxidative stress in several species are available. We investigated oxidative stress indicators as a marker for glyphosate toxicity in the liver [55,56,57]. In the present study, high amounts of LPO and TBARS were seen in glyphosate-treated animals, which might be due to hyperglycaemia. This in turn could have further accelerated the production of ROS and dysregulate glucose and fat metabolism. These oxidation processes can create free radicals, which can cause cell damage in a chain reaction. Antioxidants inhibit chain reactions by eliminating the free radical intermediates and by being oxidized themselves, they inhibit additional oxidation processes. Hence, we analyzed the levels of antioxidants in the glyphosate-treated rats in the present study.

SOD is an essential antioxidant enzyme that serves as the protective agent against ROS by scavenging superoxide radicals. It helps to prevent the development of oxygen-free radicals in tissues. The reduction in SOD activity in glyphosate-treated rat liver might have resulted in oxidative DNA or mitochondrial damage in cells. CAT is a heme protein that catalyzes the reduction of H_2_O_2_ to oxygen and water thereby protects the cell from ROS-induced toxicity caused by H_2_O_2_. It also includes Fe in its active core which shields the cell from oxidative damage [58,59]. Fe deficiency may be the cause of reduced CAT enzyme activity in the liver tissue of glyphosate-exposed rats [60]. Furthermore, through affecting insulin production and blood glucose balance in rats, glyphosate can elevate ROS production and metabolic imbalance.

Glyphosate can generate oxidative stress, which causes hyperglycaemia by stimulating the sympathetic nervous system and the hypothalamic-pituitary-adrenal (HPA) axis. As a result, glucagon, catecholamines and growth hormone are released, which boosts gluconeogenesis, glycogenolysis and insulin resistance. In this study also the mechanism by which glyphosate might have altered the glucose metabolic pathways in rats [53,61]. Hence, the activities of critical glycolytic and gluconeogenic enzymes were assessed in the present study. In glyphosate-exposed rats, glucose metabolic enzymes such as HK, PK, G6P and F1,6BP were significantly altered. The decline in the activities of HK and PK in glyphosate-treated rat liver might be attributed to reduced glycolysis and glucose uptake for oxidation [62,63]. Due to insulin and glucose intolerance, which raises lipogenesis and blood glucose levels, the glyphosate-treated groups showed significant increase in G6P and F1,6BP activity [64]. Under physiological conditions, insulin typically limits gluconeogenesis; hence, the rise in these gluconeogenic enzymes may be triggered by an issue with insulin action.

The initiation of the hepatic insulin signalling mechanism is mediated by autophosphorylation, stimulation, and activation of scaffold signalling molecules such IRS1 and IRS2 [65]. The role of both isoforms in stable glucose regulation is the same. The glyphosate-treated group in the present study had significantly lower levels of IR mRNA, is suggestive of a shift in insulin transduction. Hyperinsulinemia could have contributed to the reduced IR mRNA expression levels in the glyphosate-treated groups. As the transit of insulin from the endoplasmic reticulum (ER) to the plasma membrane requires glycines retained in the insulin receptor, a lack of insulin receptors induces hyperglycaemia and diabetes. Another factor is that cytochrome *c* oxidase (COX) is the enzyme responsible for the last step of ATP production in mitochondria. Substitutions for conserved glycines severely impede COX’s oxidative phosphorylation. This could explain that the fact that glyphosate is toxic to mitochondria [66,67]. As shown in this study, glyphosate activates cellular stress response pathways and creates ROS, which may lead to enhanced oxidation of membrane molecules. This shift in the antioxidant and oxidative stress balance damages biomolecules including DNA, proteins and lipids, leading to non-alcoholic fatty liver disease (NAFLD), which is a major contributor to hepatic insulin resistance [68,69].

An organophosphate like glyphosate, which has a phosphate group, binds with anticholinesterase (AChE) through a covalent bond between the glyphosate phosphate and the oxygen of serine at the active site of AChE. This causes an irreversible phosphorylation of inactive AChE and increases acetylcholine (Ach) activity. Extreme addition of Ach at cholinergic sites causes nicotinic effects, such as metabolic shifts, resulting in significantly increased oxygen and glucose requirements, as well as ATP requirements, throughout the muscles. As a result of the increased energy demand, NADH, which is a glycolysis pathway product, is oxidized with ambient oxygen, increasing free radical formation (ROS) and increased production of GLUT2. These ROS cause the glucose transporter to be altered in the lysosome by inducing the insulin stimulation at PI3-kinase to phosphorylate Rac GTPase. As a result, intravascular glucose and GLUT2 levels stay high, resulting in a defective GLUT2 and insulin action [70]. GLUT2 regulates the majority of glucose uptake in hepatocytes, which is influenced by the amount of circulating glucose in the bloodstream. In the current study, the GLUT2 mRNA expression was significantly amplified in the liver of glyphosate-treated group. These findings show that glyphosate disrupts glucose homeostasis through the production of ROS which leads to insulin resistance.

The primary function of hepatic insulin is to influence lipid metabolism. SREBP-1c, a transcription factor controlled by insulin, is essential for de novo lipogenesis, the process by which sugar is converted into fat. As a result, glyphosate-exposed rats exhibited elevated liver SREBP1c mRNA levels. The present work has provided a strong evidence that glyphosate alters lipid metabolism in response to oxidative stress that results in the detrimental changes on lipid markers in glyphosate treated rats [71].

Glucose homeostasis is also influenced by ligand-activated transcription factors from the peroxisome proliferator-activated receptors superfamily of nuclear hormone receptors (PPARs). In type 2 diabetes patients, PPAR-γ activation lead to a significant improvement in insulin and glucose indices, owing to an increase in whole body insulin sensitivity [72]. It plays an imperative function in the regulation of lipid metabolism in adult adipocytes by enhancing fatty acid entrapment. PPAR-γ activation has been linked to positive impact on the expression and release of a wide spectrum of cytokines and disruptions in their production may lead to metabolic disorders. The mechanisms of PPAR-mediated insulin sensitivity are complicated and particular effects on skeletal muscle, fat and liver are considered to be involved [73]. In the present study, mRNA expression of PPAR-γ in glyphosate exposed rats was found to be reduced which could be due to glyphosate induced increase in the LPO and H_2_O_2_ in the liver that leads to decreased expression of PPAR-γ compared to control rats. In accordance with present study, it has been reported that glyphosate impede the activation of PPAR-γ via enhanced lipid peroxidation, decrease preadipocyte proliferation and differentiation and thereby resulting in oxidative stress, which is suggestive of its potential to disrupt cellular physiology [74].

**Figure 13:**
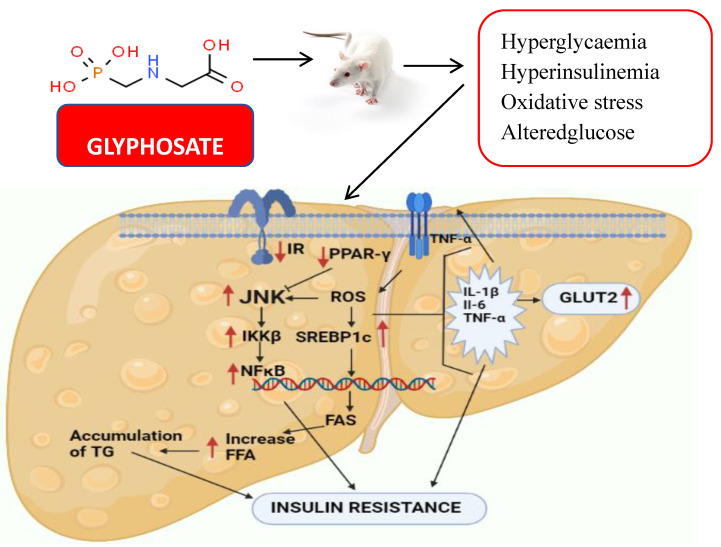
The diagram shows the effect of glyphosate on hepatic insulin signalling and inflammation in male albino rats. Glyphosate exposure results in hyperglycaemia, hyperinsulinemia and oxidative stress in rats that alter the insulin signalling in the liver. Hepatic glucose metabolism was also disturbed as a result, and pro-inflammatory cytokines were increased, leading to NAFLD and insulin resistance.

SREBP1c and PPAR-γ are known to control lipid metabolism altered in hepatotoxicity and might lead to NAFLD in response to glyphosate exposure. As they affect the stress pathway, these components may be involved in a crosstalk network with JNK and participates in metabolic a pathway that links inflammation to metabolic disorders like insulin resistance through PPAR-γ and NF-κB. JNK activation via IKKβ and NF-κB in response to pro-inflammatory indicators may also lead to disease progression in insulin resistance and atherosclerosis. In the present study, it was clearly observed that glyphosate exposure led to a significant increase in the mRNA levels of JNK, IKKβ and NFκB, as well as pro-inflammatory markers (TNF-α, IL-6 and IL-1β) when compared to that of control rats. These data indicates a shift in insulin signalling that might have led to inflammation and systemic insulin resistance [75,76]. Exposure of rats to Roundup (a herbicide containing glyphosate) caused chronic inflammation in their liver and adipose tissue as reported by Pandey et al. [77]. When adult male rats were treated with varied dosages (0, 5, 10, 25, 50, 100 and 250 mg/kg bodyweight [bw]) of Roundup, it resulted in increased levels of C-reactive protein, cytokines IL-1, TNF-, IL-6 and inflammatory response marker, as well as prostaglandin-endoperoxide synthase in the liver and adipose tissue. Along with our study, it has been shown that short-term Roundup exposure promotes liver scarring, multi-organ inflammation and liver dysfunction in adult male rats. Taken together, our present findings strongly bring experimental evidence that glyphosate exposure causes liver inflammation-induced insulin resistance in rats (Figure 13). Future research need to concentrate on understanding the genetic pathways as well as the therapeutic aspects of glyphosate-regulated insulin signalling aiding T2DM research.

## 5. Conclusions

Glyphosate aggravated ROS, altered antioxidant activity, disrupted the glucose homeostasis, altered insulin action in liver via NFκB signaling in liver. As the primary organ for metabolism and detoxification, the liver is affected by oxidative stress caused by glyphosate, which could change crucial regulatory pathways including insulin signalling and JNK, resulting in insulin resistance and inflammation. Further research is needed to understand how glyphosate affects inflammation-related glucose homeostasis, which is important for T2DM/insulin signalling.

## Figures and Tables

**Table 1:** Primer details.

**S. No.**	**Gene Details**	**Primer Details**	**Reference**
1.	GLUT2	Forward: 5′-CTC GGG CCT TAC GTG TTC TTC CTT-3′ Reverse: 5′-TGG TTC CCT TCT GGT CTG TTC CTG-3′	[37]
2.	IR	Forward: 5′-GCC ATC CCG AAA GCG AAG ATC-3′ Reverse: 5′-TCT GGG GAG TCC TGA TTG CAT-3′	[37]
3.	SREBP1c	Forward: 5′-GGA GCC ATG GAT TGC ACA TT-3′ Reverse: 5′-GCT TCC AGA GAG GAG CCC AG-3′	[38]
4.	PPAR-γ	Forward: 5′-CCT GAA GCT CCA AGA ATA CC-3′ Reverse: 5′-GAT GCT TTA TCC CCA CAG AC-3′	[39]
5.	NFκB	Forward: 5′-CAT GAA GAG AAG ACA CTG ACC ATG GAA A-3′ Reverse: 5′-TGG ATA GAG GCT AAG TGT AGA CAC G-3′	[40]
6.	JNK	Forward: 5′-TCA GAA TCC GAA CGA GAC AAA AT-3′ Reverse: 5′-AAG CCA GAG TCC TTC ACA GAC AA-3′	[41]
7.	IL-6	Forward: 5′-GTG AGA AGT ATG AGA AGT GTG A-3′ Reverse: 5′-GCA GGA TGA GAA TGA TCT TTG-3′	[42]
8.	IKKβ	Forward: 5′-TGG CAT GGA AAC GGA TAA CTG A-3′ Reverse: 5′-CTG GAA CTC TGT GCC TGT GGA A-3′	[43]
9.	TNF-α	Forward: 5′-GTC GTA GCA AAC CAC CAA GC-3′ Reverse: 5′-TGT GGG TGA GGA GCA CAT AG-3′	[44]
10.	IL-1β	Forward: 5′- GCA ATG GTC GGG ACA TAG TT-3′ Reverse: 5′-AGA CCT GAC TTG GCA GAG A-3′	[44]
11.	β-actin	Forward: 5′-AAG TCC CTC ACC CTC CCA AAA G-3′ Reverse: 5′-AAG CAA TGC TGT CAC CTT CCC-3′	[35]

**Table 2:** OGT in glyphosate exposed diabetic animals.

Groups	0 h	60 min	120 min	180 min
Control	69 ± 1.2	72 ±1.4	75 ±3.2	76 ± 4.2
Glyphosate (50 mg)	110 ± 1.5 ^a^	115 ± 2.2 ^a^	120 ± 3.5 ^a^	121 ± 3.4 ^a^
Glyphosate (100 mg)	132 ± 2.5 ^ab^	138 ± 5.9 ^ab^	149 ± 6.2 ^ab^	150 ± 6.2 ^ab^
Glyphosate (250 mg)	138 ± 0.5 ^ab^	140 ± 6.7 ^ab^	152 ± 7.8 ^ab^	159 ± 7.7 ^ab^

The effect of glyphosate on OGT in control and glyphosate treated rats. Data were expressed as mean ± SEM where *n* = 6 rats for each group. Values significant at *p* < 0.05 were analyzed with ^a^—control, ^b^—glyphosate (50 mg/kg b.wt.).

**Table 3:** Level insulin tolerance in glyphosate exposed animals.

Group	0 h	15 min	30 min	45 min	60 min
Control	68 ± 2.9	70 ±4.2	62 ±1.2	69 ±3.2	70 ±2.9
Glyphosate (50 mg)	109 ± 7.29 ^a^	110 ± 6.29 ^a^	109 ±4.2 ^a^	110 ±4.9 ^a^	101 ±5.2 ^a^
Glyphosate (100 mg)	129 ± 1.9 ^a^	117 ± 1.5 ^a^	116 ±5.2 ^ab^	110 ±6.7 ^a^	112 ± 6.5 ^a^
Glyphosate (250 mg)	127 ± 6.2 ^b^	122 ±5.9 ^ab^	129 ±6.2 ^ab^	127 ±7.9 ^ab^	112 ±6.4 ^a^

The effect of glyphosate on insulin tolerance in control and glyphosate treated rats. Data were expressed as mean ± SEM where *n* = 6 rats for each group. Values significant at *p* < 0.05 were analyzed with ^a^—control, ^b^—glyphosate (50 mg/kg b.wt.).

**Table 4:** Levels of HOMA-IR and QUICKI in glyphosate explore diabetic rats.

Groups	HOMA-IR	QUICKI
Control	2.76 ± 0.11	0.92 ± 0.05
Glyphosate (50 mg)	9 ± 0.52 ^a^	0.8 ± 0.03 ^a^
Glyphosate (100 mg)	10± 0.42 ^a^	0.62 ± 0.02 ^ab^
Glyphosate (250 mg)	12± 0.25 ^ab^	0.6 ± 0.03 ^ab^

The effect of glyphosate on HOMA-IR and QUICKI in control and glyphosate treated rats. Data were expressed as mean ± SEM where *n* = 6 rats for each group. Values significant at *p <* 0.05 were analyzed with ^a^—control, ^b^—glyphosate (50 mg/kg b.wt.).

## Data Availability

The data presented in this study are available in this article.

## References

[1] Saeedi P., Petersohn I., Salpea P., Malanda B., Karuranga S., Unwin N., Colagiuri S., Guariguata L., Motala A.A., Ogurtsova K. (2019). Global and regional diabetes prevalence estimates for 2019 and projections for 2030 and 2045: Results from the International Diabetes Federation Diabetes Atlas. IDF Diabetes Atlas Committee (2019), 9th ed. Diabetes Res. Clin. Pract..

[2] Bonciu E. (2012). Cytological effects induced by Agil herbicide to onion. J. Hortic. For. Biotechnol..

[3] Mesi A., Kopliku D., Golemi S. (2012). The use of higher plants as bio-indicators of environmental pollution—A new approach for toxicity screening in Albania. Mediterr. J. Soc. Sci..

[4] Silveira M.A., Ribeiro D.L., Dos Santos T.A., Vieira G.M., Cechinato C.N., Kazanovski M., Grégio d’Arce L.P. (2016). Mutagenicity of two herbicides widely used on soybean crops by the Allium cepa test. Cytotechnology.

[5] Kraehmer H., van Almsick A., Beffa R., Dietrich H., Eckes P., Hacker E., Hain R., Strek H.J., Stuebler H., Willms L. (2014). Herbicides as weed control agents: State of the art: II. Recent achievements. Plant Physiol..

[6] Nicolopoulou-Stamati P., Maipas S., Kotampasi C., Stamatis P., Hens L. (2016). Chemical Pesticides and Human Health: The Urgent Need for a New Concept in Agriculture. Front. Public Health.

[7] Cai D., Yuan M., Frantz D.F., Melendez P.A., Hansen L., Lee J., Shoelson S.E. (2005). Local and systemic insulin resistance resulting from hepatic activation of IKK-beta and NF-kappaB. Nat. Med..

[8] He Z., King G.L. (2004). Microvascular complications of diabetes. Endocrinol. Metab. Clin. N. Am..

[9] Zambrano S., De Toma I., Piffer A., Bianchi M.E., Agresti A. (2016). NF-κB oscillations translate into functionally related patterns of gene expression. eLife.

[10] Rothschild D., Weissbrod O., Barkan E., Kurilshikov A., Korem T., Zeevi D., Costea P.I., Godneva A., Kalka I.N., Bar N. (2018). Environment dominates over host genetics in shaping human gut microbiota. Nature.

[11] Komives T., Schroder P. (2016). On glyphosate. Ecocycles.

[12] Thongprakaisang S., Thiantanawat A., Rangkadilok N., Suriyo T., Satayavivad J. (2013). Glyphosate induces human breast cancer cells growth via estrogen receptors. Food Chem. Toxicol. Int. J. Publ. Br. Ind. Biol. Res. Assoc..

[13] Silva V., Montanarella L., Jones A., Fernández-Ugalde O., Mol H., Ritsema C.J., Geissen V. (2018). Distribution of glyphosate and aminomethylphosphonic acid (AMPA) in agricultural topsoils of the European Union. Sci. Total Environ..

[14] Mertens M., Höss S., Neumann G., Afzal J., Reichenbecher W. (2018). Glyphosate, a chelating agent-relevant for ecological risk assessment?. Environ. Sci. Pollut. Res. Int..

[15] Williams G.M., Berry C., Burns M., de Camargo J.L., Greim H. (2016). Glyphosate rodent carcinogenicity bioassay expert panel review. Crit. Rev. Toxicol..

[16] Kapoor D., Malkin C.J., Channer K.S., Jones T.H. (2005). Androgens, insulin resistance and vascular disease in men. Clin. Endocrinol..

[17] Andersson B., Mårin P., Lissner L., Vermeulen A., Björntorp P. (1994). Testosterone concentrations in women and men with NIDDM. Diabetes Care.

[18] Muthusamy T., Dhevika S., Murugesan P., Balasubramanian K. (2007). Testosterone deficiency impairs glucose oxidation through defective insulin and its receptor gene expression in target tissues of adult male rats. Life Sci..

[19] Vandenberg L.N., Blumberg B., Antoniou M.N., Benbrook C.M., Carroll L., Colborn T., Everett L.G., Hansen M., Landrigan P.J., Lanphear B.P. (2017). Is it time to reassess current safety standards for glyphosate-based herbicides?. J. Epidemiol. Community Health.

[20] Nardi J., Moras P.B., Koeppe C., Dallegrave E., Leal M.B., Rossato-Grando L.G. (2017). Prepubertal subchronic exposure to soy milk and glyphosate leads to endocrine disruption. Food Chem. Toxicol. Int. J. Publ. Br. Ind. Biol. Res. Assoc..

[21] Martinez A., Al-Ahmad A.J. (2019). Effects of glyphosate and aminomethylphosphonic acid on an isogeneic model of the human blood-brain barrier. Toxicol. Lett..

[22] Trasande L., Aldana S.I., Trachtman H., Kannan K., Morrison D., Christakis D.A., Whitlock K., Messito M.J., Gross R.S., Karthikraj R. (2020). Glyphosate exposures and kidney injury biomarkers in infants and young children. Environ. Pollut..

[23] Pandey A., Rudraiah M. (2015). Analysis of endocrine disruption effect of Roundup® in adrenal gland of male rats. Toxicol. Rep..

[24] Liu J.B., Li Z.F., Lu L., Wang Z.Y., Wang L. (2022). Glyphosate damages blood-testis barrier via NOX1-triggered oxidative stress in rats: Long-term exposure as a potential risk for male reproductive health. Environ. Int..

[25] Matthews D.R., Hosker J.P., Rudenski A.S., Naylor B.A., Treacher D.F., Turner R.C. (1985). Homeostasis model assessment: Insulin resistance and beta-cell function from fasting plasma glucose and insulin concentrations in man. Diabetologia.

[26] Katz A., Nambi S.S., Mather K., Baron A.D., Follmann D.A., Sullivan G., Quon M.J. (2000). Quantitative insulin sensitivity check index: A simple, accurate method for assessing insulin sensitivity in humans. J. Clin. Endocrinol. Metab..

[27] Fraga C.G., Leibovitz B.E., Tappel A.L. (1988). Lipid peroxidation measured as thiobarbituric acid-reactive substances in tissue slices: Characterization and comparison with homogenates and microsomes. Free. Radic. Biol. Med..

[28] Jiang Z.Y., Hunt J.V., Wolff S.P. (1992). Ferrous ion oxidation in the presence of xylenol orange for detection of lipid hydroperoxide in low density lipoprotein. Anal. Biochem..

[29] Kakkar P., Das B., Viswanathan P.N. (1984). A modified spectrophotometric assay of superoxide dismutase. Indian J. Biochem. Biophys..

[30] Sinha A.K. (1972). Colorimetric assay of catalase. Anal. Biochem..

[31] Brandstrup N., Kirk J.E., Bruni C. (1957). The hexokinase and phosphoglucoisomerase activities of aortic and pulmonary artery tissue in individuals of various ages. J. Gerontol..

[32] Valentine W.N., Tanaka K.R. (1966). Pyruvate kinase: Clinical aspects. Methods Enzym..

[33] Koide H., Oda T. (1959). Pathological occurrence of glucose-6-phosphatase in serum in liver diseases. Clin. Chim. Acta.

[34] Gancedo J.M., Gancedo C. (1971). Fructose-1,6-diphosphatase, phosphofructokinase and glucose-6-phosphate dehydrogenase from fermenting and non fermenting yeasts. Arch. Mikrobiol..

[35] Fourney R.M., Day M.J. (1988). Randall RJRS. Northern blotting: Efficient RNA staining and transfer. Focus.

[36] Jayashree S., Indumathi D., Akilavalli N., Sathish S., Selvaraj J., Balasubramanian K. (2013). Effect of Bisphenol-A on insulin signal transduction and glucose oxidation in liver of adult male albino rat. Environ. Toxicol. Pharmacol..

[37] Bizeau M.E., MacLean P.S., Johnson G.C., Wei Y. (2003). Skeletal Muscle Sterol Regulatory Element Binding Protein-1c Decreases with Food Deprivation and Increases with Feeding in Rats. J. Nutr..

[38] Mahmoud A.M., Abdel-Rahman M.M., Bastawy N.A., Eissa H.M. (2017). Modulatory effect of berberine on adipose tissue PPAR, adipocytokines and oxidative stress in high fat diet/streptozotocin-induced diabetic rats. J. Appl. Pharm. Sci..

[39] Al-Rasheed N.M., Fadda L.M., Al-Rasheed N.M., Ali H.M., Yacoub H.I. (2016). Down-Regulation of NFκB, Bax, TGF-α, Smad-2mRNA expression in the Livers of Carbon Tetrachloride Treated Rats using Different Natural Antioxidants. Braz. Arch. Biol. Technol..

[40] Zhou H., Li Y.J., Wang M., Zhang L.H., Guo B.Y., Zhao Z.S., Meng F.L., Deng Y.G., Wang R.Y. (2011). Involvement of RhoA/ROCK in myocardial fibrosis in a rat model of type 2 diabetes. Acta Pharmacol. Sin..

[41] Lu L., Zhang Q., Pu L.J., Xu X.W., Zhang R.Y., Zhang J.S., Hu J., Yang Z.K., Lü A.K., Ding F.H. (2007). Elevation of tumor necrosis factor-alpha, interleukin-1beta and interleukin-6 levels in aortic intima of Chinese Guizhou minipigs with streptozotocin-induced diabetes. Chin. Med. J..

[42] Qiu L.L., Wang C., Yao S., Li N., Hu Y., Yu Y., Xia R., Zhu J., Ji M., Zhang Z. (2019). Fenvalerate induces oxidative hepatic lesions through an overload of intracellular calcium triggered by the ERK/IKK/NF-κB pathway. FASEB J..

[43] Dange R.B., Agarwal D., Teruyama R., Francis J. (2015). Toll-like receptor 4 inhibition within the paraventricular nucleus attenuates blood pressure and inflammatory response in a genetic model of hypertension. J. Neuroinflamm..

[44] Peinnequin A., Mouret C., Birot O., Alonso A., Mathieu J., Clarençon D., Agay D., Chancerelle Y., Multon E. (2004). Rat proinflammatory cytokine and cytokine related mRNA quantification by real-time polymerase chain reaction using SYBR green. BMC Immunol..

[45] Gabe M. (1968). Techniques Histologiques.

[46] Gill J.P.K., Sethi N., Mohan A., Datta S., Girdhar M. (2018). Glyphosate toxicity for animals. Environ. Chem. Lett..

[47] Mesnage R., Renney G., Séralini G.E., Ward M., Antoniou M.N. (2017). Multiomics reveal non-alcoholic fatty liver disease in rats following chronic exposure to an ultra-low dose of Roundup herbicide. Sci. Rep..

[48] Samuel V.T., Shulman G.I. (2016). The pathogenesis of insulin resistance: Integrating signaling pathways and substrate flux. J. Clin. Investig..

[49] Dallegrave E., Mantese F.D., Oliveira R.T., Andrade A.J., Dalsenter P.R., Langeloh A. (2007). Pre-and postnatal toxicity of the commercial glyphosate formulation in Wistar rats. Arch. Toxicol..

[50] Romano R.M., Romano M.A., Bernardi M.M., Furtado P.V., Oliveira C.A.D. (2010). Prepubertal exposure to commercial formulation of the herbicide glyphosate alters testosterone levels and testicular morphology. Arch. Toxicol..

[51] Mechanick J.I. (2006). Metabolic mechanisms of stress hyperglycemia. JPEN J. Parenter. Enteral. Nutr..

[52] Tizhe E., Ibrahim N., Fatihu M., Ambali S., Igbokwe I., Tizhe U. (2018). Pancreatic function and histoarchitecture in Wistar rats following chronic exposure to Bushfire^®^: The mitigating role of zinc. J. Int. Med. Res..

[53] Rajesh P., Sathish S., Srinivasan C., Selvaraj J., Balasubramanian K. (2013). Phthalate is associated with insulin resistance in adipose tissue of male rat: Role of antioxidant vitamins. J. Cell. Biochem..

[54] Ponnulakshmi R., Shyamaladevi B., Vijayalakshmi P., Selvaraj J. (2019). In silico and in vivo analysis to identify the antidiabetic activity of beta sitosterol in adipose tissue of high fat diet and sucrose induced type-2 diabetic experimental rats. Toxicol. Mech. Methods.

[55] Bagchi D., Bagchi M., Hassoun E.A., Stohs S.J. (1995). In vitro and in vivo generation of reactive oxygen species, DNA damage and lactate dehydrogenase leakage by selected pesticides. Toxicology.

[56] Palmeira C.M., Moreno A.J., Madeira V.M. (1995). Thiols metabolism is altered by the herbicides paraquat, dinoseb and 2,4-D: A study in isolated hepatocytes. Toxicol. Lett..

[57] Milić M., Žunec S., Micek V., Kašuba V., Mikolić A., Lovaković B.T., Semren T.Ž., Pavičić I., Čermak A., Pizent A. (2018). Oxidative stress, cholinesterase activity, and DNA damage in the liver, whole blood, and plasma of Wistar rats following a 28-day exposure to glyphosate. Arh. Hig. Rada Toksikol..

[58] Rikans L.E., Yamano T. (2000). Mechanisms of cadmium mediated acute hepatotoxicity. J. Biochem. Mol. Toxicol..

[59] Chance B., Greenstein D.S., Roughton R.J.W. (1952). The mechanism of catalase action 1-steady state analysis. Arch. Biochem. Biophys..

[60] Jurczuk M., Brzoska M.M., Moniuszko-Jakoniuk J., Galazyn-Sidorczuk M., Kulikowska-Karpinska E. (2004). Antioxidant enzymes activity and lipid peroxidation in liver and kidney of rats exposed to cadmium and ethanol. Food Chem. Toxicol..

[61] Djeffal A., Messarah M., Boumendjel A., Kadeche L., Feki A.E. (2015). Protective effects of vitamin C and selenium supplementation on methomyl-induced tissue oxidative stress in adult rats. Toxicol. Ind. Health.

[62] Baquer N.Z., Gupta D., Raju J. (1998). Regulation of metabolic pathways in liver and kidney during experimental diabetes: Effects of antidiabetic compounds. Indian J. Clin. Biochem..

[63] Vats V., Yadav S.P., Grover J.K. (2003). Effect of *T. foenumgraecum* on glycogen content of tissues and the key enzymes of carbohydrate metabolism. J. Ethnopharmacol..

[64] Chakrabarti S., Biswas T.K., Rokeya B., Ali L., Mosihuzzaman M., Nahar N., Khan A.K., Mukherjee B. (2003). Advanced studies on the hypoglycemic effect of Caesalpinia bonducella F. in type 1 and 2 diabetes in Long Evans rats. J. Ethnopharmacol..

[65] Mugabo Y., Lim G.E. (2018). Scaffold proteins: From coordinating signaling pathways to metabolic regulation. Endocrinology.

[66] Uren Webster T.M., Santos E.M. (2015). Global transcriptomic profiling demonstrates induction of oxidative stress and of compensatory cellular stress responses in brown trout exposed to glyphosate and Roundup. BMC Genom..

[67] da Silva Rosa S.C., Nayak N., Caymo A.M., Gordon J.W. (2020). Mechanisms of muscle insulin resistance and the cross-talk with liver and adipose tissue. Physiol. Rep..

[68] Hamdaoui L., Naifar M., Mzid M., Ben Salem M., Chtourou A., Ayedi F., Sahnoun Z., Rebai T. (2016). Nephrotoxicity of Kalach 360 SL: Biochemical and histopathological findings. Toxicol. Mech. Methods.

[69] Yazdinezhad A., Abbasian M., Hojjat Hosseini S., Naserzadeh P., Agh-Atabay A.H., Hosseini M.J. (2017). Protective effects of Ziziphora tenuior extract against chlorpyrifos induced liver and lung toxicity in rat: Mechanistic approaches in subchronic study. Environ. Toxicol..

[70] Namba T., Nolte C.T., Jackrel J., Grob D. (1971). Poisoning due to organophosphate insecticides: Acute and chronic manifestations. Am. J. Med..

[71] Ren X., Dai P., Perveen A., Tang Q., Zhao L., Jia X., Li Y., Li C. (2019). Effects of chronic glyphosate exposure to pregnant mice on hepatic lipid metabolism in offspring. Environ. Pollut..

[72] Blaschke F., Takata Y., Caglayan E., Law R.E., Hsueh W.A. (2006). Obesity, peroxisome proliferator-activated receptor, and atherosclerosis in type 2 diabetes. Arter. Thromb Vasc. Biol.

[73] Leonardini A., Laviola L., Perrini S., Natalicchio A., Giorgino F. (2009). Cross-Talk between PPARgamma and Insulin Signaling and Modulation of Insulin Sensitivity. PPAR Res..

[74] Martini C.N., Gabrielli M., Brandani J.N., Vila Mdel C. (2016). Glyphosate Inhibits PPAR Gamma Induction and Differentiation of Preadipocytes and is able to Induce Oxidative Stress. J. Biochem. Mol. Toxicol..

[75] Jayaraman S., Devarajan N., Rajagopal P., Babu S., Ganesan S.K., Veeraraghavan V.P., Palanisamy C.P., Cui B., Periyasamy V., Chandrasekar K. (2021). β-Sitosterol Circumvents Obesity Induced Inflammation and Insulin Resistance by down-Regulating IKKβ/NF-κB and JNK Signaling Pathway in Adipocytes of Type 2 Diabetic Rats. Molecules.

[76] Prasad M., Jayaraman S., Rajagopal P., Veeraraghavan V.P., Kumar P.K., Piramanayagam S., Pari L. (2022). Diosgenin inhibits ER stress-induced inflammation in aorta via iRhom2/TACE mediated signaling in experimental diabetic rats: An in vivo and in silico approach. Chem.-Biol. Interact..

[77] Pandey A., Dhabade P., Kumarasamy A. (2019). Inflammatory Effects of Subacute Exposure of Roundup in Rat Liver and Adipose Tissue. Dose-Response A Publ. Int. Hormesis Soc..

